# Corrigendum to A multimodal strategy to improve health care for pediatric patients with cancer and fever in Peru

**DOI:** 10.26633/RPSP.2023.163

**Published:** 2023-11-13

The *Pan American Journal of Public Health* draws readers’ attention to an error in the following article, pointed out by the authors:

In page 4, figure 1 the word Lorem ipsum should be Vancomycin

**FIGURE 1.**
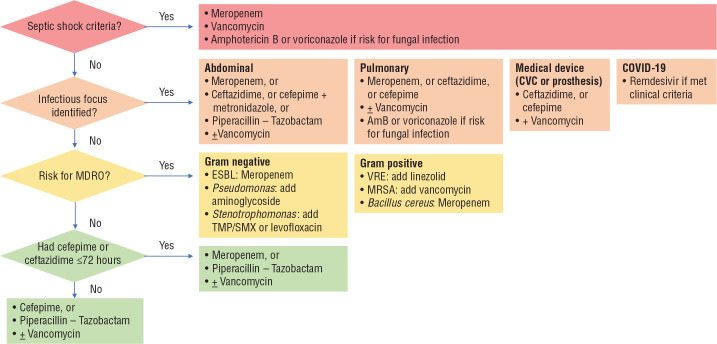
DoTT project antibiotic selection pathways

***Note:*** AmB, amphotericin B; CVC, central venous catheter; DoTT, Decreasing Time to Therapy; ESBL, extended spectrum beta lactamase; MRSA, methicillin resistant *Staphylococcus aureus*; TMP/SMX, trimethoprim/sulfamethoxazole; VRE, vancomycin resistant enterococcus.

***Source:*** Prepared by the authors.

